# Immune dysregulation in Kabuki syndrome: a case report of Evans syndrome and hypogammaglobulinemia

**DOI:** 10.3389/fped.2023.1087002

**Published:** 2023-06-09

**Authors:** Lucia Leonardi, Alessia Testa, Mariavittoria Feleppa, Roberto Paparella, Francesca Conti, Antonio Marzollo, Alberto Spalice, Fiorina Giona, Maria Gnazzo, Gian Marco Andreoli, Francesco Costantino, Luigi Tarani

**Affiliations:** ^1^ Department of Maternal Infantile and Urological Sciences, Sapienza University of Rome, Rome, Italy; ^2^ Pediatric Unit, IRCCS Azienda Ospedaliero-Universitaria di Bologna, Bologna, Italy; ^3^ Pediatric Hematology, Oncology and Stem Cell Transplant Division, Padua University Hospital, Padua, Italy; ^4^ Department of Translational and Precision Medicine, Sapienza University of Rome, Rome, Italy; ^5^ Translational Cytogenomics Research Unit, Bambino Gesù Children’s Hospital, IRCCS, Rome, Italy

**Keywords:** Kabuki syndrome, Evans syndrome, autoimmunity, immunodeficiency, hypogammaglobulinemia, immune dysregulation

## Abstract

Kabuki syndrome (KS) is a rare multisystemic disease due to mutations in the *KMT2D* or *KDM6A* genes, which act as epigenetic modulators of different processes, including immune response. The syndrome is characterized by anomalies in multiple organ systems, and it is associated with autoimmune and inflammatory disorders, and an underlying immunological phenotype characterized by immunodeficiency and immune dysregulation. Up to 17% of KS patients present with immune thrombocytopenia characterized by a severe, chronic or relapsing course, and often associated to other hematological autoimmune diseases including autoimmune hemolytic anemia, eventually resulting in Evans syndrome (ES). A 23-year-old woman, clinically diagnosed with KS and presenting from the age of 3 years with ES was referred to the Rare Diseases Centre of our Pediatric Department for corticosteroid-induced hyperglycemia. Several ES relapses and recurrent respiratory infections in the previous years were reported. Severe hypogammaglobulinemia, splenomegaly and signs of chronic lung inflammation were diagnosed only at the time of our observation. Supportive treatment with amoxicillin-clavulanate prophylaxis and recombinant human hyaluronidase-facilitated subcutaneous immunoglobulin replacement were immediately started. In KS patients, the failure of B-cell development and the lack of autoreactive immune cells suppression can lead to immunodeficiency and autoimmunity that may be undiagnosed for a long time. Our patient's case is paradigmatic since she presented with preventable morbidity and severe lung disease years after disease onset. This case emphasizes the importance of suspecting immune dysregulation in KS. Pathogenesis and immunological complications of KS are discussed. Moreover, the need to perform immunologic evaluations is highlighted both at the time of KS diagnosis and during disease follow-up, in order to allow proper treatment while intercepting avoidable morbidity in these patients.

## Introduction

Kabuki syndrome (KS) is a rare, multiple congenital anomaly/intellectual disability syndrome with an estimated prevalence of 1:32,000 in Japan (1), where it was first described in 1981. The prevalence outside Japan is presumably similar to that seen in the Japanese population, but is not known (2). In 2010, Ng et al. identified heterozygous mutations in *KMT2D* as the main genetic cause of KS (3). More recently, mutations in *KDM6A* have been reported in almost 5% of KS cases. However, genetic basis of the syndrome is still unknown in up to 20%–25% of the patients (4). Both *KMT2D*, inherited as autosomal dominant, and *KDM6A*, inherited as X-linked, are involved in embryogenesis, development and immune response, functioning as epigenetic modulators explaining the characteristic phenotype of KS patients (5, 6).

Immune abnormalities may occur later in childhood, being characterized by immune dysregulation with increased risk of autoimmune diseases and immunodeficiency and, therefore, of infections (7). Among autoimmune diseases, immune thrombocytopenic purpura (ITP), hemolytic anemia [often combined in Evans syndrome (ES)], thyroiditis and vitiligo are reported (8–10). Autoimmune cytopenia associated with KS usually present with a chronic and relapsing course and a poor response to conventional therapy (11–14). Non-malignant lymphoproliferation has also been described as a feature of KS, similarly to other inborn errors of immunity (15, 16).

We report the case of a 23-year-old woman with KS and a history of several relapses of ES, referred to the Rare Diseases Centre of our Pediatric Department for corticosteroid-induced hyperglycemia. Severe hypogammaglobulinemia was also diagnosed at the time of our first observation.

## Case description

**Figure 1:**
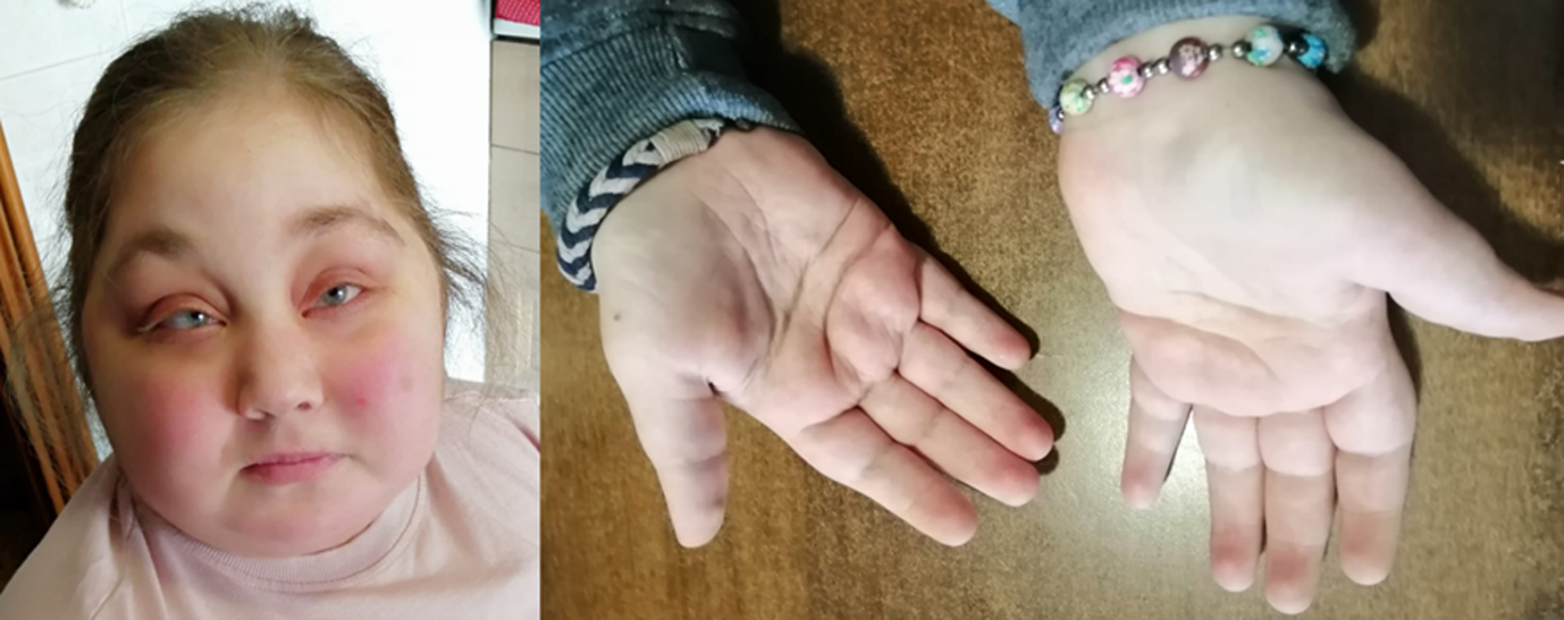
Patient's facial features (arched and broad eyebrows, long palpebral fissures, eversion of the lower eyelid, depressed nasal tip and short columella) and persistence of fetal fingertip pads, typical of Kabuki syndrome.

A 23-year-old woman, clinically diagnosed with KS at the age of 5 years, was referred to our Rare Diseases Centre for hyperglycemia following high-dose prednisone treatment due to an ES relapse. The patient was born at term from cesarean section due to fetal distress. She was the third daughter of healthy Italian non-consanguineous parents, with no family history of immunological, autoimmune or rare diseases. Neonatal examination highlighted bifid uvula. At the age of three, she was diagnosed with autoimmune hemolytic anemia (AIHA) with a positive direct antiglobulin test, needing long-term corticosteroid therapy. During infancy she also presented with several episodes of sinusitis, otitis media and three episodes of pneumonia that required hospitalization. Moreover, she was diagnosed with ostium secundum atrial septal defect, surgically corrected at the age of 5 years. During hospitalization, genetic counseling highlighted the presence of major criteria for clinical diagnosis of KS including moderate intellectual disability, postnatal short stature, skeletal abnormalities, dermatoglyphic anomalies and facial dysmorphism (Figure 1).

Shortly afterward, the patient presented with ITP which, associated with AIHA, led to ES diagnosis. Over the years, the patient underwent several courses of corticosteroids due to ES relapses; however, since the age of 12, she had been poorly followed in any aspect of her syndrome except for neuromuscular rehabilitation.

At the time of our observation, at the age of 23 years, the patient presented with a peculiar KS facial appearance, short stature, obesity (BMI 43 kg/m^2^), mild developmental delay, multiple skeletal defects (dorsal kyphosis, lumbar hyperlordosis, bilateral pronated feet) and dermatoglyphic anomalies. Moreover, she presented with clinical side effects of systemic glucocorticoids therapy including *Striae rubrae*, central obesity, and arterial hypertension. Severe hyperglycemia (glucose level >400 mg/dl) with elevated HbA1C level (>9%) led to glucocorticoid-induced diabetes mellitus diagnosis. GAD65, ICA and IA-2A autoantibodies were not detected. Serum glucose concentration had always been lower than 160 mg/dl before prednisone treatment.

Short-term glycemic control with a target range of 100–180 mg/dl throughout the day was chosen. Routine blood glucose monitoring revealed a typical pattern of steroid-induced diabetes characterized by near-normal fasting glucose levels followed by hyperglycemia during the day. Standard dietary counseling and short-acting insulin were therefore initiated.

**Figure 2:**
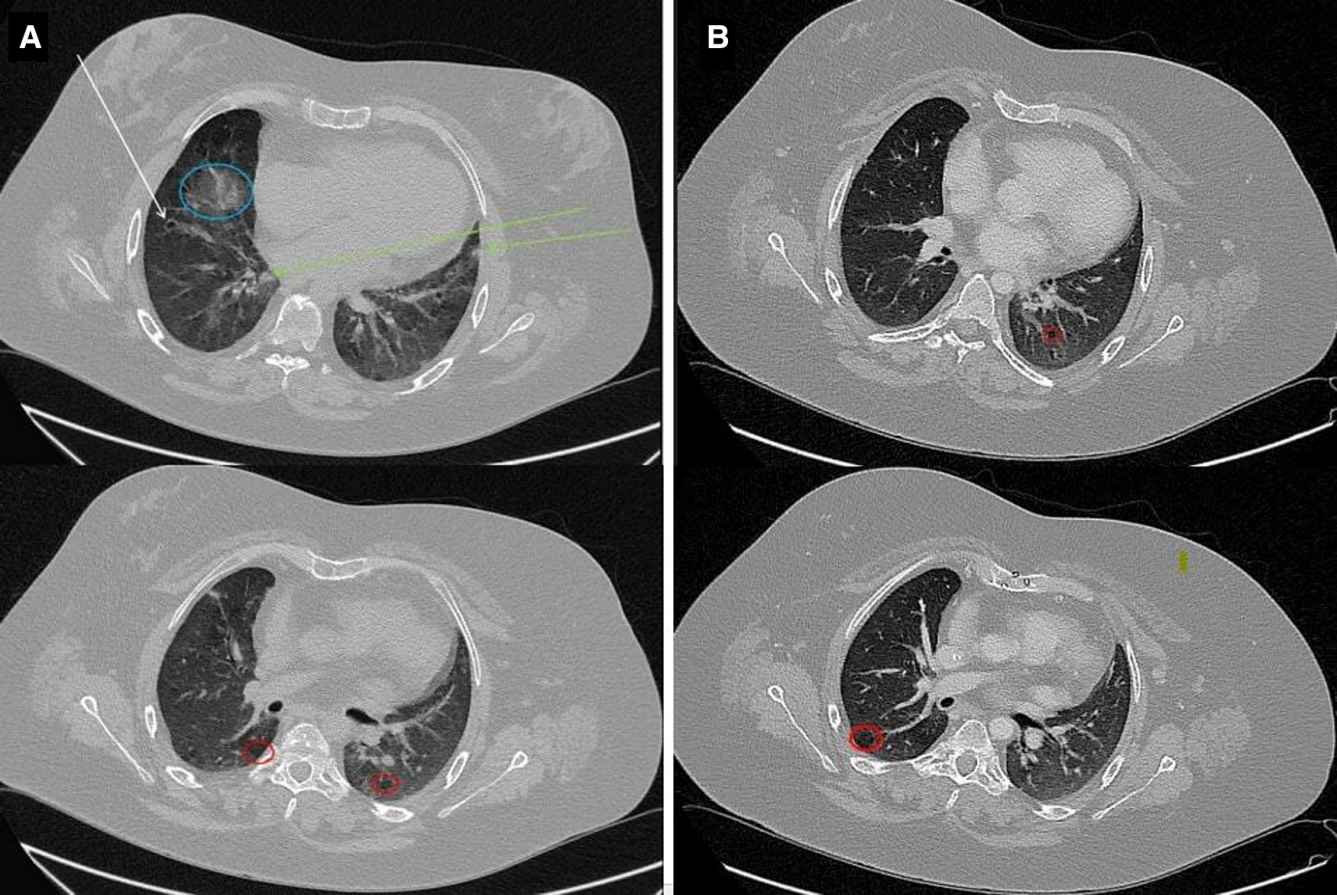
Chest HRCT. (**A**) Images show interstitial lung disease and chronic inflammation. Note the numerous findings, including ground-glass opacity (blue circle), bronchiectasis (white arrow), nodules with blurred edges (green arrows), and regions of air trapping (red circles). (**B**) Radiological disease improvement with persistence of air-trapping.

Further investigations indicated mild bilateral sensorineural hearing loss, renal anomalies and alternating strabismus. Echocardiography documented bilateral ventricular wall thickening, first-degree diastolic dysfunction of the left ventricle, and right ventricular systolic function below normal range. Abdominal ultrasound showed hepatic steatosis, mild splenomegaly (spleen longitudinal diameter = 12.6 cm), and bicornuate uterus. High-resolution computed tomography (HRCT) of the lungs revealed diffuse ground-glass opacities, interlobular septal and bronchial wall thickening, subsolid nodules with blurred edges, peribronchial calcifications and low-attenuation areas due to air trapping (Figure 2A). These findings were interpreted as interstitial lung disease and chronic inflammation, likely due to both recurrent respiratory infections and immune dysregulation, while monoclonal lymphoproliferative disorders were excluded. Given the diagnosis of ES, the chronic lung disease and the splenomegaly, an immunological evaluation was performed highlighting severe hypogammaglobulinemia: IgG 200 mg/dl (reference value 700–1,600); IgA 2 mg/dl (reference value 68–400) and IgM 149 mg/dl (reference value 40–259). T-cell phenotype showed: CD3^+^ = 574 cells/µl (CD4^+ ^= 298 cells/µl, 29%; CD8^+^ = 219 cells/µl, 21%). B- cell phenotype was characterized by a normal circulating B-cell number (CD19^+^ B-cells = 364 cells/µl), with a reduction in IgD^+^CD27^+^ memory (3%) and IgD^−^CD27^+^ switched memory (2.5%) B-cells percentage, as well as an expanded population of CD21^low^ B-cells (12%). Lymphocyte level ranged between 700 and 1100 cells/µl in sequential analyses. Antibiotic prophylaxis with amoxicillin-clavulanate and replacement treatment with recombinant human hyaluronidase-facilitated subcutaneous immunoglobulin (fSCIg) were initiated, therefore limiting further immunological studies. With regard to the lung imaging, although solid data regarding diagnostic and prognostic markers of granulomatous -lymphocytic interstitial lung disease (GLILD) are currently lacking, and considerably variable histopathological findings in GLILD patients have been described, the diagnostic hypothesis of GLILD was considered. Lung biopsy and bronchoscopy, however, were not performed because of the high risk of the procedure due to disease severity and comorbidities, while lung diffusion testing was limited by the patient's poor compliance.

Genetic testing was conducted in accordance with the Helsinki Declaration, after obtaining informed consent from the patient's parents. Sequencing analysis for Kabuki syndrome panel detected a nonsense variant in the *KMT2D* (NM_003482.3) gene: c.8974G>T, p.Glu2992Ter. This variant, confirmed by Sanger analysis, was not reported in the database of human variations GnomAD (https://gnomad.broadinstitute.org/) and was classified as likely pathogenic according to the American College of Medical Genetics and Genomics (ACMG) criteria (17).

**Figure 3:**
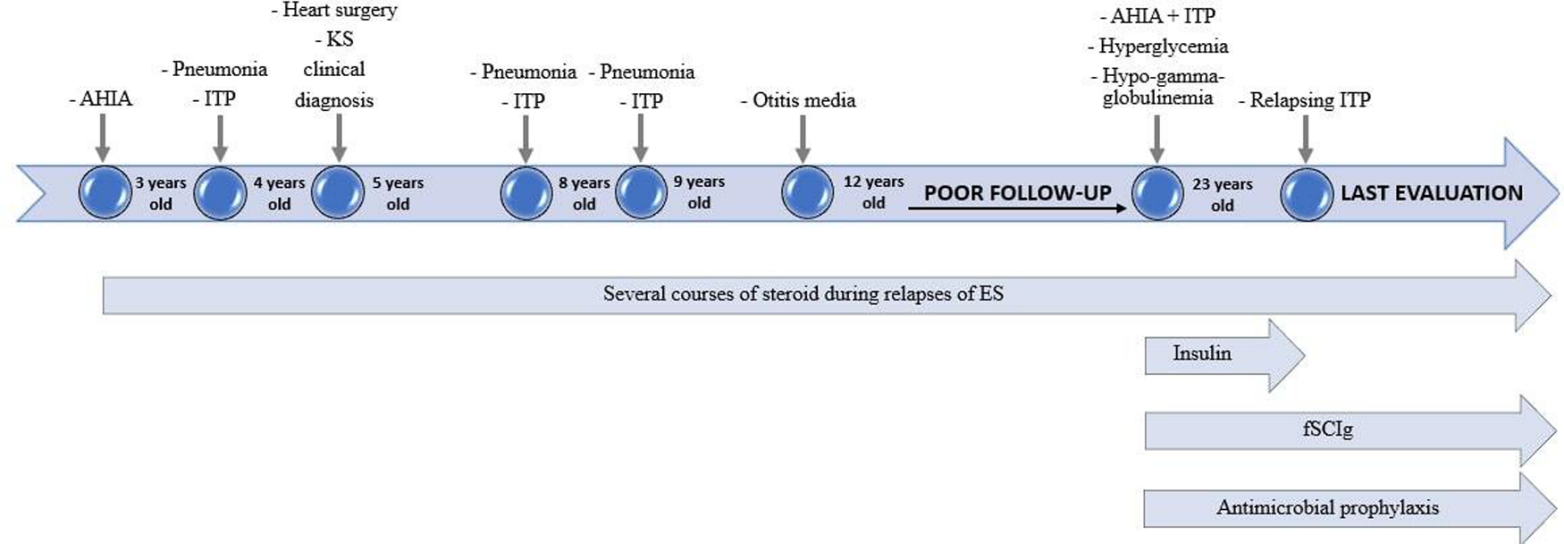
Timeline of principal clinical events and therapeutic strategies.

At 6-month follow-up evaluation after fSCIg and antibiotic initiation, no infections were reported. Bimonthly fSCIg have been administered at the dosage of 0.4 g/kg. The frequency of infusions was adjusted according to IgG levels and clinical history over time. Pre-infusion IgG levels of 700–800 mg/dl, associated to an optimal clinical outcome, were finally reached with a monthly fSCIg replacement dosage of 0.4 g/kg. Insulin was no longer needed since blood glucose target concentration was reached only by diet. HRCT of the lungs performed during follow-up demonstrated an important decrease of the previously described opacities and nodules, with the sole persistence of air-trapping (Figure 2B). Considering the radiological disease improvement obtained with immunoglobulin replacement, the diagnosis of GLILD was eventually ruled out. Hemoglobin level remained at about 12 emsp14;g/dl in the absence of any transfusion and without features of hemolysis. ITP, which initially was partially responsive to high-dose prednisone treatment, improved after immunoglobulin replacement, with a platelet count durably greater than 70,000/µL; relapses, previously documented at any attempt of corticosteroid withdrawal, became fewer and better controlled with low-dose corticosteroids (Figure 3). Alternative therapies, including rituximab have been therefore postponed.

## Discussion

We hereby report the case of a 23-year-old woman affected by KS presenting with hypogammaglobulinemia, chronic lung disease due to recurrent respiratory infections and immune dysregulation, and several relapses of ES progressively increasing in frequency despite corticosteroid courses.

An international consensus in 2019 defined KS diagnostic criteria (18, 19). The syndrome is characterized by dysmorphic facies (defined by an eversion of the lower lateral eyelids, arched eyebrows with sparse lateral part, depressed nasal tip and prominent ears), postnatal hypotonia, growth deficiency, several skeletal and visceral malformation (including cleft palate, congenital heart defects), dermatoglyphic anomalies and mild to moderate grade of intellectual impairment. Beyond multiorgan anomalies and intellectual disability, autoimmune disorders, including ITP, AIHA, vitiligo and thyroiditis may complicate KS presentation. In KS patients, ITP seems to be the most frequent among immune-mediated cytopenias, but cases of AIHA and/or neutropenia, either combined with ITP or presenting as single-line cytopenia, are also described (7, 12, 20).

Primary autoimmune cytopenias in childhood, especially ITP, are often self-limiting disorders or responsive to first-line therapy (21). Conversely, early-onset, long-lasting, multilineage, refractory cytopenias should strongly suggest an underlying monogenic immune defect (20, 22–24). Consistently with the abovementioned findings, when associated with KS, autoimmune cytopenias, and more specifically ITP, are characterized by severe presentation with chronic or relapsing symptoms, poorly responsive to conventional treatments (13). Moreover, in several patients with KS, non-malignant lymphoproliferation and recurrent respiratory infections, due to hypogammaglobulinemia, have been reported (25, 26).

KS is indeed a paradigmatic example of immunological dysregulation due to mutation in the *KMT2D* or *KDM6A* genes, involved in epigenetic modulation of immune system function. The *KMT2D* gene, also known as *MLL2*, encodes for a histone H3 lysine 4 (H3K4) mono-methyltransferase, essential for cell differentiation and embryonic development (27). Methylation on H3K4 is found in actively transcribed genes. The *KDM6A* gene, also known as *UTX*, encodes for the lysine-specific demethylase 6A linked with demethylation of lysine residues on histone, in particular H3K27, resulting in a gene de-repression. Methylation on H3K27 is associated with transcriptional repression, therefore the action of the *KDM6A* product allows chromatin opening and active gene transcription (28). Mutations of these enzymes cause an impairment of epigenetic activation of certain genes, leading to the distinctive developmental abnormalities of KS. The immune characteristics of KS patients with *KMT2D* mutations may derive from a loss of H3K4 methylation at crucial transcription factors, that finally dysregulates B and T differentiation. A disrupted terminal B-cell differentiation and an impaired somatic immunoglobulin hypermutation have been observed in patients with *KMT2D* mutations, likely as result of impaired epigenetic regulation of the IGH locus. KS patients can in fact present with hypogammaglobulinemia and reduced number of memory B-cells associated to expansion of CD21 B-cell population (29).

Moreover, a clustering of missense mutations in the terminal region of the *KMT2D* gene might increase the risk for autoimmune diseases, depending either on defective regulatory T-cells (Tregs) generation or intrinsic B tolerance breakage (10, 12, 29, 30). Immunodeficiency may worsen with age and more than 80% of KS patients, mostly with *KMT2D* mutations, display hypogammaglobulinemia and diminished memory B-cell populations (7). However, autoimmune diseases or hypogammaglobulinemia may not occur until later in childhood, being often underestimated or undiagnosed for a long time, leading to a substantial diagnostic delay. Our patient's case is paradigmatic, since she was referred to our center several years after the onset of refractory autoimmune disease, while also presenting with severe lung disease, suggesting a long-term, undiagnosed, hypogammaglobulinemia. Immunological study was indeed performed for the first time in this patient in occasion of our first observation.

Susceptibility to infections, especially of middle ear and upper respiratory tract, is common among patients with KS (31). Hearing loss, mainly due to recurrent middle ear infections, occurs approximately in up to 80% of patients, and can be conductive, sensorineural or mixed (1, 32, 33). An early immunoglobulin replacement therapy could therefore have a positive effect on hearing function by preventing recurrent otitis media. It is thus advisable to perform a routine and periodic immunologic evaluation in KS patients, in order to prevent chronic diseases and to reduce morbidity and mortality rate. Moreover, because most of KS cases are reported in the pediatric age, the actual frequency of immune alterations in adults with KS may be underestimated.

In addition, as aforementioned, autoimmune cytopenias in KS are severe and often refractory to conventional treatment with corticosteroids. Thus alternative therapies, including rituximab, sirolimus and mycophenolate mofetil (13, 15, 34) should be considered. Moreover, although glucocorticoid-induced diabetes mellitus generally resolves when corticosteroids are discontinued, periodic blood glucose assessment should always be performed in case of long-term steroid treatment (35) to prevent complications, such as diabetic ketoacidosis, due to the impairment of pancreatic endocrine function. In this scenario, multidisciplinary evaluation coordinated by a case manager and immunological evaluations during follow-up are crucial. In our case, patient's overall clinical condition improved after fSCIg replacement initiation. No infections, as well as a lower recurrence of AIHA and ITP episodes were observed. In conclusion, this case highlights the need of improving awareness in suspecting immune dysregulation in KS patients. Moreover, it is advisable that KS patients undergo immunologic evaluations at diagnosis and during follow-up, aiming to provide adequate treatment and avoid preventable and severe morbidity.

## Data Availability

The datasets presented in this study can be found in online repositories. The names of the repository/repositories and accession number(s) can be found below: https://databases.lovd.nl/shared/variants/0000881406#00023885, 0000881406.
